# Understanding women’s decision-making process for birth location in Tanzania based on individual women’s reproductive pathways: a life-course perspective

**DOI:** 10.1080/16549716.2022.2040149

**Published:** 2022-03-24

**Authors:** Andrea Solnes Miltenburg, Sandra van Pelt, Benedikte Lindskog, Johanne Sundby, Tarek Meguid

**Affiliations:** a Department of Obstetrics and Gynaecology, Akershus University Hospital, Lørenskog, Norway; b Institute of Health and Society, Department of Community Medicine and Global Health, Faculty of Medicine, University of Oslo, Oslo, Norway; c Department of Work and Social Psychology, Maastricht University, Maastricht, The Netherlands; d Section for Diversity Studies Department of International Studies and Interpreting OsloMet, Oslo Metropolitan University, Oslo, Norway; e Child Health Unit. University of Cape Town, South Africa

**Keywords:** Life course, care-seeking, maternal health

## Abstract

**Background:**

Determinants for women’s care seeking for birth in low-income setting are multifactorial and remain poorly understood. A life course approach can assist to structure the interplay of the different factors that lead to women seeking care or not.

**Objective:**

In this study we aimed to explore individual women’s reproductive pathways, and increase understanding of how important life events including previous pregnancy and birth experiences can help us to understand individual choices made for care seeking during childbirth.

**Methods:**

The study took place in Tanzania between 2015 and 2017. 14 women were followed throughout their pregnancy, birth and postpartum period through participant observation and in-depth interviews. In total 94 in-depth interviews were held (between 5–7 interviews per woman). Analysis occurred continuous throughout the data collection period resulting in detailed narratives of crucial events across women’s life course, with specific attention to their current pregnancy.

**Results:**

Of the 14 women, seven had a facility birth, six a home birth and one woman gave birth at the home of a local birth attendant. Four different story plots were identified: expected home birth, expected facility birth, unexpected facility birth and unexpected home birth. Birth narratives of four women representative of the different story plots are presented. Narratives illustrate women’s individual reproductive pathways including the various factors influencing women’s expectations and justifications for their actions during their pregnancy and birth.

**Conclusion:**

Women’s agency, including women’s perception of self, the self in relation to the social environment and reflection on risks associated with the range of options, influences the final decision made for birth. Women’s narratives suggest that quality of care can function as a primary pull factor for facility birth. As long as home birth is by some perceived to be a better alternative, achieving skilled care for all will be difficult to achieve.

## Background

Birth with a skilled birth attendant (SBA) has been a measurable target in the effort to reduce maternal mortality, and globally, more women are giving birth in facilities [1]. Although poor quality of care may compromise the significance of the policy, promoting facility birth remains an important global objective. It aims to ensure that all women can give birth in safe environments, with skilled providers that have the tools and competencies to care for women during uncomplicated birth and who can detect and deal with complications if and when they occur [2,3]. Many countries in sub-Saharan Africa, including Tanzania, have been successful in increasing both the availability of and access to health facilities, which has led to more women seeking care during pregnancy [4]. Nearly all women in Tanzania attend to at least one antenatal care (ANC) visit indicating that women are able to get to health facilities and receive services, if they are provided during daytime hours and on a scheduled basis [5]. However, similar success has never been achieved for coverage of facility births. Many women still give birth at home (37%), despite being able to access health facilities during pregnancy [5].

Factors that influence women’s decision to seek care during birth are considered to be multifactorial, influenced by continuously changing social circumstances [6,7]. Attempting to understand health service use has resulted in the development of several models, of which the health belief model and socio-behavioural model are most commonly known. These models have been revised intensely in recent years [8,9]. Both models describe facilitators and barriers to care seeking, including environmental or external factors (e.g. socio-demographic, health system, cultural and community factors) and individual level factors (illness perspective or perceived need) [8,9]. Within maternal healthcare, studies report similar determinants for care seeking, and give specific attention to additional factors, which include: women’s perception of pregnancy and birth; women’s perception and previous experience with the available quality of care; women’s status in society and their socio-cultural context and; available resources and access to care [6,7]. Studies on maternal mortality in Tanzania frequently refer to the ‘three delays model’ [10] with delays in care seeking for birth – 1) the decision to seek care; 2) reaching an adequate healthcare facility and 3) in receiving adequate care at the facility. Delays occur in one or all phases of delay. It remains poorly understood *why* and *how* Tanzanian women weigh the range of known factors in their ultimate decision for birth location [7].

Health decision-making is a complex process where individuals move through a series of stages and phases. At each stage, where interactions and events at each of these stages influences their choices and behaviours [9]. Scholars who emphasize a life-course perspective in their research understand decision making through an implicit ‘theory of action’ [11]. A life-course perspective sees each person as an ‘individual behavioural system’, with personal values, social expectations and motives, who is adapting to their environment. A person’s life-path, including decisions made to go in one direction or another, are the result of several key internal and external factors [11]. This paper uses the life-course approach to situate Tanzanian women’s decision making for birth in the context of space and time. In Tanzania, a young woman’s first pregnancy greatly influences what happens next in her life [12]. Pregnancy brings on different responsibilities. For many women, their first individual interactions with the health system and societal expectation of their role as a mother-to-be. Rather than approaching every pregnancy as one isolated event, each pregnancy should be seen in the context of interrelated dynamic socio, economic and environmental factors, which influence subsequent pregnancies, providing women and their social networks with important cumulative experiences.

Drawing on ethnographic research amongst pregnant women in Tanzania and through the collection of narratives/life stories, we aimed to increase our understanding of how important life events during women’s reproductive pathways can help to understand choices made for care seeking during pregnancy and birth.

## Life-course approach for maternal health

A life-course approach has become a powerful organizing framework for the study of the determinants of health, primarily in epidemiology, allowing for understanding of early development processes and environmental influences on disease and mortality over time [13]. The life-course approach extends across disciplines in social, behavioural and biological sciences and has allowed for the study of the influence of social change and life transitions on individual behaviour [14]. Within sexual and reproductive health, the life-course includes the various stages of life (pre-pregnancy, pregnancy, neonatal period, infancy and childhood, adolescence, and into the post-reproduction stage) and explores how conditions in the different stages influence health conditions later in life [15]. For this study we zoom in on a specific period of the reproductive stages, notably women’s lives from adolescence to the various pregnancies that followed. We follow four paradigmatic factors of the life-course approach according to Elder [16]: 1) the interplay of human lives in historical times, 2) the timing of lives, 3) interdependent lives and 4) human agency in choice making [16].

### The interplay of human lives in historical times

The period or time in which people are born provides a geographical, cultural and historical meaning that influences certain life opportunities, which can be similar for women born in the same birth cohort [14]. Women that currently are in their reproductive age were born between the year 1970–2000, starting their reproductive lives from the early 1980s and onwards. During this time important global transitions within the field of health occurred, including an emphasis on women’s health, with particular attention to the high rates of maternal mortality in sub-Saharan Africa. This attention led to the launch of the Safe Motherhood Initiative in 1987 and continued focus on maternal health came with the Millennium Development Goals in 2000 [17]. Global recommendations for birth changed from accepting home birth with traditional birth attendants, as the norm in the 1980s, to primary focus on ensuring facility birth for all from the year 2000 onwards [18].

### The timing of lives

The timing of certain life events, for example marriage and childbearing, influences women’s lives [14]. *When* these life events occur can be early or late relative to other people and normative expectations. Marriage and leaving their paternal home are important life transitions for women. Some women are subjected to arranged marriages and the payment of dowry is a common practice in many countries in sub-Saharan Africa. The age at which girls and young women marry can determine whether they manage to complete their education or not. Childbearing and becoming a mother are significant milestones for women providing them with an important social identity [19]. On the other hand, not being able to bear children can cause a significant burden on them [20].

### Interdependent lives

The lives of individuals are interwoven with the lives of others. Lives change as relationships and social roles change [14]. The meaning and influence of family relations and social ties depends on the cultural and historical context. The role of individuals in relation to their family can be considered quite different between and within countries. The interdependence of people on one another and the role of community is, in many countries in sub-Saharan Africa, often more important than the primary value of individual autonomy [21]. The influence of social networks, including marital status, therefore plays an important role in women’s care seeking behaviour [10,22]. For example, the influence of women’s partners on decision-making and ensuring the availability of funds for supplies, transport and emergency expenses [6,7].

### Human agency in choice making

Although the concept of ‘agency’ is defined differently across disciplines, it broadly covers an understanding of ‘individual choice’ and ‘capacity to act’ [23]. Humans hold the capacity for self-directed action, but individuals differ in their understanding of their own abilities and capacities [23]. Both this perception and the actual capacity to act, are socially influenced and can be stimulated or suppressed depending on the type and timing of situations. For women, this perception of their own self-efficacy determines if women feel the power to influence their situations, and can both aid and hinder decision-making. Women that experience a strong sense of self-efficacy have the potential to anticipate different success scenarios, are able to persevere in the face of obstacles, and are capable of taking action against ruling social norms [24]. Women exert agency through their selection or construction of the environment. For example, people tend to avoid activities and situations they believe exceed their coping abilities but they readily select social environments they judge themselves capable of handling [24].

Through a life-course perspective we aimed at increasing understanding of how perceptions of risk and opportunities in relation to ‘the self’ influences women’s decision to seek care, or not, from one pregnancy to the next. Frequent and recurrent interactions with the health system over time have an important role to play because these shape women’s expectations and risks perception. In this study, we aimed to explore individual women’s reproductive pathways and to identify how significant life events can help us to understand individual choices made for care seeking during childbirth.

## Methods

### Study setting

The study took place from September 2015 to February 2017 in a rural district in Mwanza region in the Lake Zone of Tanzania. The Sukuma ethnic group constitute 90% of the population in this region and are considered the largest of Tanzania’s more than 120 different ethnic groups. The majority of the population in Mwanza region live in rural villages where the economy is mainly based on agricultural production, livestock keeping and fishing. An abolishment of primary school fees between 2002 and 2006 resulted in an increase in girls’ enrolment, and the majority of primary-aged boys and girls now attend primary school [25].

### Data collection

By using qualitative methods, we aimed to capture individual behaviour that is not easily captured by quantitative methods. Quantitative measurements can potentially overlook the context-specific, individual, cultural, sociopolitical, economic and environmental factors that influence health and well-being throughout the life-course [11]. Qualitative methods, such as in-depth interviews, conversations and observations allow for a nuanced understanding of the contextual and cultural factors that shape healthcare practices through the use of multiple qualitative methods over an extended time period [26]. For this study, the data was collected using multiple in-depth interviews and observations of care.

ASM and SP followed 14 women throughout their pregnancy, birth and in the post-partum period through observations of the care they received and by performing multiple interviews with women over time. In total, 94 in-depth interviews were conducted, scheduled within one or two weeks of women’s clinic visits and after birth (between 5–7 interviews per woman). Interviews lasted between 1–3 hours and took place at the woman’s home, or at a location of their choosing. Interviews were performed in Kiswahili and a translator assisted where necessary. All interviews were recorded. Due to the nature of the narrative approach, interviews were guided by some general topics of interests, but relied mainly on women’s reports and personal accounts. Therefore, every interview started with a focus on description of, and reflection on the previous ANC visit, or birth experience (whether at home or in the health facility). At times, we prompted women based on their ANC card (record) with documentation of the services received, or based on our own observations. Over time, as women became more familiar with us, we discussed their reproductive history, starting with their first pregnancy experience.

During the data collection period, we spent a total of 52 days at the ANC clinic or maternity ward at the three health facilities. Observation reports were written after each day. From the start of the project until 2017, women were purposively recruited during observations at the ANC clinics where initial contact was established. Women’s permission for participation in the study was requested during the first interview at the woman’s house. Women were recruited using a staggered approach at selected health facilities to ensure researchers did not follow more than four women at the same time. A total of six women that were approached during their ANC visits were unable to be located for follow-up interview after two attempts. One woman refused to participate and one woman was lost for follow-up after moving to another district. After inclusion in the study we scheduled our observations at the antenatal clinics to match with the women’s scheduled clinic visits. In total, 25 antenatal visits were observed. Of the 7 women that gave birth at the health facility, we observed one woman through her entire birth, one woman we only saw during the admission process and another woman requested that ASM assist her during her birth at the facility. During our visits at the health facilities we observed and participated in care where possible to assist the nurse/midwives. Analysis of the quality of care women received has been published elsewhere [27,28].

### Narrative analysis

Analysing observed and recorded data occurred continuously throughout the data collection period by ASM and SP. Full observation reports were written after each observation day. In addition, personal reflections, including feelings and interpretation of the activities, were written down in the same reports or in personal diaries. All interviews were recorded and transcribed in Kiswahili and then translated into English by a research assistant. Transcripts and observation reports were used to develop a coherent narrative for each woman’s full reproductive pathway. Development of these narratives resulted from an iterative process, whereby we returned to women to ask for clarification or further exploration of discussed events. Narrative theory, as a methodological framework, is useful for a life course perspective as narratives allow for ‘preserving of the complexities of human action with its relationship of temporal sequence, human motivation, chance happenings, and changing interpersonal and environmental context’ [29]. The narrative is event centred, located in a particular time and space [30], focused on crucial events across a woman’s life course and specifically related to the woman’s current pregnancy. Through recurrent dialogue with the women, we placed these events in chronological order developing narratives with a beginning, middle and end. ‘Emplotment’ of events allows for linking of live events, explaining the end or outcome of the storyline through relating these to critical moments of human action, intention and how these instigate responses in others [30,31]. The plot was focused on the outcome of the current pregnancy and whether the woman gave birth at home or in the health facility.

A thematic analysis was performed to illuminate commonalities and differences across women’s narratives with regard to women’s decision-making processes. ASM identified main themes and subthemes in the transcripts and notes made from observations. These were sorted for amongst other live events, facilitators and barriers to care seeking and other related factors influencing decision making. We performed an in-depth narrative analysis on the identified themes of interest. Such an in-depth narrative analysis helps to reveal how people do things, how decisions were made, how choices determined actions and how these instigated responses in people’s surroundings. It allowed for exploring ‘the complex motives that drive individuals to act in some ways, rather than others and they also reveal the constraints of environment, of body, of social contexts that limit a person’s possibilities for action [30].’ Interpretation of the narratives required a deeper understanding of the events described and their meaning within the social and cultural environment that shaped the story [32]. Therefore, after all women had given birth and narratives were written, we analysed the narratives through discussion with a local expert team. The team included a Tanzanian obstetrician/gynaecologist, a midwife and a young mother with both positive and negative birth experiences. Women’s expectations, justifications for their actions and choices around previous and current pregnancies in relation to important life events, helped to understand and give meaning to the eventual location of birth.

Through the analysis of all birth narratives we identified four different story plots: an expected home birth, an expected facility birth, an unexpected facility birth and an unexpected home birth. We expected a woman’s birth location based on our regular interactions with them, their expression of plans to give birth at home or at the health facility, whether they made certain preparations for their birth or based on their perceived ability to seek care or not.

### Researcher positioning/reflexivity

‘Reflexivity’ is a process where researchers acknowledge that their perspectives ‘are shaped by their socio-historical locations, including the values and interests that these locations confer upon them’ [33]. ASM (a medical doctor) and SP (a registered nurse) performed the data collection for this study. Both authors (coming from Europe) speak Kiswahili and were familiar with the study area after spending several years working for a community-based project in that area and volunteering at different health facilities. ASM has a Tanzanian husband and was pregnant herself during data collection in 2016, which influenced the interactions with women and health staff. The pregnancy gave a certain legitimacy to ask women more personal questions and discuss bodily experiences and rationales for care seeking. Repeat visits to women’s homes increased familiarity, confidence and mutual trust. Women increasingly shared personal details they initially had left out and seemed to offer less socially desirable answers as time went on. Through reflexivity, accounting for effects the authors had on the research and data material, it contributed to distinguish between knowledge formed from preconceptions and new knowledge emerging from the research inquiry [34].

### Validity and trustworthiness

Trustworthiness can be demonstrated through an account of the process of *reflexivity* and *transferability* [34,35]. We ensured trustworthiness by employing several strategies to remedy potential bias and minimize possible validity threats [36]. SP and ASM were able to build positive relations with the women and health providers in this study thanks to their long-term involvement. This allowed us to observe women’s actions, thereby not relying exclusively on their reported behavior and thoughts. Holding between 5 to 7 interviews with the same woman allowed us to revisit previously discussed issues, gain clarification and further explore questions that arose during the writing of each of their narratives. Additionally, time between the interviews allowed the researchers and the women to reflect and deepen the subsequent discussions. We were able to conduct many of the interviews in Kiswahili and this helped to remove the challenges of working with a translator, including the risk of losing the meaning of what was being said. Including women in our study with a variety of backgrounds, allowed for reviewing discrepancies and comparisons between the women’s background characteristics, their experiences and choices made. Finally, by conducting data collection with two researchers our findings and continuous personal reflections supplemented and contested each other, allowing for deepened understanding of the complex phenomena of health decision making [34].

## Results

All 14 women had a vaginal birth. Seven women gave birth in a health facility, six at home and one at the home of a local birth attendant. Characteristics of the individual women are presented in Table 1. Four women had an expected facility birth, three an unexpected facility birth, five women had an expected home birth [including one woman giving birth with a local birth attendant], and two women had an unexpected home birth.

**Table 1.** Overview of individual characteristics, health care seeking behaviour and outcome

Name*	Years in school	Work	Marital status	SES**	Age first birth	Previous birth location	Facility distance	No of ANC visits	Quality score of ANC ****	Decision making for birth	Location of birth	Newborn outcome
Rory (22y) G2P1	11	Yes	Married	3	18	1F0H	1–5 km	4	6,25	Expected FB	District Hospital	Alive
Diana (30y) G4P2Φ	7	Yes	Married	4	22	2F0H	<1 km	5	8,40	Expected FB	District Hospital	Alive
Jessica (25y) G5P4	6	No	Married	2	16	1F3H	5–10 km	5	6,10	Unexpected FB	District Hospital	Alive
Angel (22y) G2P1	7	No	Married	3	17	1F0H	1–5 km	3	3,50	Expected FB	Health Centre	Stillbirth
Flora (21y) G2P1Φ	7	No	Married	1	19	0F1H	5–10 km	4	6,25	Unexpected FB	Health Centre	Infant died at 3 months
Jane (18y) G1P0	9	No	Relationship	3	-	-	1–5 km	3	6,67	Expected FB	District Hospital***	Infant died at 6 months
Tara (37y) G7P6	5	No	Relationship	2	16	5F1H	<1 km	2	NA	Unexpected FB	Health Centre	Alive
Maria (22y) G2P1Φ	7	Yes	Relationship	3	14	0F1H	<1 km	2	5,50	Unexpected HB	Home	Alive
Bea (29y) G4P2	12	Yes	Married	4	24	2F0H	1–5 km	3	4,17	Unexpected HB	Home	Alive
Pili (19y) G2P1	7	No	Married	3	17	1F0H	>10 km	4	5,17	Expected HB	Home	Alive
Naima (19y) G3P1	7	No	Married	1	16	1F0H	>10 km	3	3,49	Expected HB	TBA home	Alive
Mariam(32y) G6P6	7	No	Married	2	22	3F2H	1–5 km	4	3,67	Expected HB	Home	Alive
Paulina (37y) G8P7Φ	5	No	Married	1	16	3F4H	>10 km	5	6,80	Expected HB	Home	Alive
Helena (31y) G5P4	4	No	Married	3	17	2F4H	1–5 km	3	6,50	Expected HB	Home	Alive

G = gravidity, P = Parity, ANC = Antenatal Care, No = number, SES = socio economic status,

*Names are pseudonyms. Φ Women of whom the narratives are presented in the results

**All women are poor, but category for Socio Economic Status is determined based on a number of indicators including possession of assets, living conditions and personal background. Category levels range from very poor category 1 to more well off category 4.

***She was transferred from the health centre to the district hospital with the ambulance.

****Score out of a max score of 11 for 11 specific services (Correct determination gestational age, fundal height fetal heart rate, blood pressure measurement and urine test at each visit, HIV test at first visit, Hemoglobine and syphilis test at any visit. Provision of antimalaria and iron supplements at each visit, provision of mebendazole at any visit).

In the following paragraphs, we present narratives of four women to represent each of the four story plots we analysed of all women. Flora and Paulina^1^^1^Names are pseudonyms. were both born in poverty and married into poverty. They both lived in remote villages and relied on farming to secure enough food at home. They received financial support from their husbands, but this was inconsistent and unpredictable. Diana and Maria both lived in the town centre of the district, close to the district hospital. Although they had very different lives, they both had considerably more resources and reliable social networks than Paulina and Flora and both also had their own income.

## Paulina (Expected home birth)

The first time I (ASM) saw Paulina was mid-February, when I joined the ANC clinic. Julia, the nurse, asked me to help her with the clinic for pregnant women so she could focus on finishing the vaccinations. The woman I checked was Paulina, and this was her first ANC visit for this pregnancy. Around 1.5 weeks later we went to Paulina’s house. Paulina lives approximately a 30 minute drive from the health centre, close to the main rough road. When we approached her house, she was working on the land behind the house. She came walking towards us and welcomed us.

Paulina said she was born in 1978 in a neighbouring district. She had completed five years of schooling and had to stop school because she became pregnant in 1994. She was 16 years old at that time. She met her then ‘husband^2^^2^They refer to husbands when talking about men who they have lived with or have children with.’ Abdallah at school. When her parents found out she was pregnant, she initially denied it.

With the first one, my parents shocked me; ‘you are pregnant, right?’ I said; ’no’. ‘Speak up’! Then I said, yes, I agreed, only because I was scared, I didn’t know.

Paulina and Abdallah lived together for one year until he died in a car accident. At the time of fieldwork, Paulina’s first born, Ali, was living with his grandfather. After her first husband died she stayed with her family for a year until she met her current husband, Marco. She met him in Mwanza and he comes from the same village where they live. When she got pregnant in 1996, she moved in with him and once she moved in, she found him with his first wife, who had not yet given him children. Marco’s first wife moved out soon after Paulina started to live there. Marco never paid a dowry to her family but as she was having children with him, her father said it was good for her to live with Marco. Marco is not often at home, and during the time I got to know Paulina, it became clear that he likely had another family at the lake side. Later on, he appeared to have ‘married’ another young girl in the village. Paulina said he stays mostly at the lake side because he is a fisherman.

He usually comes, then after 2 or 3 days he leaves. That’s how it goes. He goes, he stays even 2 weeks, one week, and then he comes again. He even sleeps here several days, he leaves, he goes.

Since Paulina has had many pregnancies we had to go through all of her pregnancies over the course of several interviews. She has given birth to 7 children, all boys, but one died before he turned one year old. The first two births were in a health facility, of which the second one was not a good experience.

There was a time when I went to give birth at (large hospital), the nurses just left us, we gave birth by ourselves. Then I said; Mm, so going to hospital to give birth has no meaning, even at home I can just give birth. Completely alone. When the child fell out we called ‘nurse’ and then she came running. They took the child and then checked him.

The following five births where at home, with the exception of one. Her home births were all good experiences for her, even though she did have some complications during her fifth birth.

When I got tested (during ANC) the nurse told me to come and give birth in the hospital. ‘If you don’t come you’ll lose a lot of blood’ And really, when I didn’t go there was a lot of blood.

She had given birth at home even though the nurse had warned her. The placenta came out normally but blood kept running out. In two hours, she had to change three Kitenge (local fabric) as they were all full of blood. They called a doctor from the health centre who lives close by. He came to give her an injection, which helped to stop the bleeding. She paid him 5000Tsh (*$*2.5 USD). Despite this experience, she felt comfortable at home for the following two births.

Paulina started to open up about how she felt about her husband and how challenging it is for her. Paulina complained that her husband does not help her at all. She just tries not to depend on him if she can. She does her own little business from the house – selling soaps, cooking oil, groundnuts to people along the road. One day her husband was violent against her and she had had enough of it. She said:

He beat me, when he beat me I ran, at night. He slapped me once, I fell, when I woke up, I took my clothes, I packed them and I went! I went to the homesteads. I went to sleep there. […] The next day in the morning I went to my mother, I stayed there, then he came to make a scene there […] and then I brought him to the (village) chairperson. Yes, the chairperson said; ‘go back home and don’t beat again, don’t do it again’. I had decided not to go back anymore, I had decided to go back home (to her parents). He (the chairperson) said; ‘no, go back, it will bring problems to the children, just stay […]. Then I just went back. I continued to stay.

Paulina was diagnosed with HIV during her last pregnancy and was by now accustomed to both the reality of being HIV positive and the required treatment. During her current pregnancy, her HIV medication was changed, which caused her severe physical discomfort, she eventually stopped taking the treatment because the nurse/midwife at the ANC clinic refused to provide her with her old medication.

This medication they gave me now for this pregnancy brings me problems. When you take it you feel the earth is turning, your head is painful, the belly hurts, even until the child moves. When I started with this medication I couldn’t even go out. I was really sick inside (the house). […]I went back there [to the health centre] on Monday. […] I went back there to explain the nurse. The nurse explained me, she stated that it is not possible to change the medication, there’s only this one. The nurse refused. She said maybe you go and check for malaria at the (other) dispensary. And then you come back to tell us whether you have malaria […]. I didn’t ask (why), when they refused me, that nurse, I left. When I don’t take this medication I just feel good. […] And the way I understood, we were told ‘if the medication does not agree with you, come and they’ll change it for you’. That’s why I came back again. The nurse says, there’s no medication at all, it is really hard to change the medication.

It was not until she was attended by a known and friendly nurse/midwife at one of her last visits that her complains were taken seriously and her medication was changed. As a sign of gratitude, Paulina gave her a chicken.

I found her, I told her. She told me: ‘There are no medications to exchange. Sit here and explain me now what is bothering you’. So, I started to tell her now. I explained her and then later she said: ‘Well sit here, I’ll get the medication for you’. I sat, I waited for the medication. She gives me the normal one now.

Around her due date she called me to say she had delivered, a baby girl, safely, at home. When we visited her, I congratulated her and asked how she was feeling and how her labour started and progressed.

It (labour pains) was there for many days […] a full week […] coming and going. […] Aa! The pains started in the evening; I was called by my sister-in-law, there in the surroundings of (names village name), that Saturday. I left here (home) around 1pm, we went there, we arrived around 3pm. (We) went by bike. That boy, my child, that big one, he rode (the bike). We stayed; we talked there up till around 5pm (and then started the journey home). Aa! I saw I couldn’t stay on the bicycle anymore. I told him (her son who was driving the bicycle); just wait, I take a motorcycle, you just come with the bicycle, I took the motorcycle and then it brought me here, at home around 7pm. Then I saw that the time, to go to (health facility) and the transport fee, it’s a lot! These days, to just go to (health facility) is 4000 Tsh ($2 USD). If you don’t have, that’s it. Then I thought: the transport fee that I have, it’s better to use it for other expenses. I just stayed home, whatever would happen would happen now. We cooked here, the food was ready and we ate. Then I went to sleep. The others didn’t know. I just knew it myself, they didn’t know, nor did I tell them. They didn’t know, until it was 4am, then I woke them up and told them to go and wake up that neighbour. […] They went to call that neighbour, when she came I had already given birth. There (pointing at the room where she gave birth), on the floor. I arranged clothes and I placed a bag, that’s it. […] I just laid down flat. When I felt the contraction I pushed, I pushed like that, I pushed once, I felt the child was coming. I pushed again, I pushed twice, three times, I then took the child out. When I gave birth, I stayed just a bit […] I pressed the belly, then everything (the placenta) came out.

I asked if she had a lot of blood loss this time, she said it was not much but, perhaps two pieces of kitenge full. She had taken some local medicine, which had helped her to stop the bleeding.

## Diana (Expected facility birth)

I (SP) met Diana at the end of October when she was seated in the waiting area of the ANC clinic. It was around 8am and she was waiting for the clinic to start. After her visit, I observed her ANC card and saw that it was written that she was 12 + 6 weeks pregnant, noticing she started early with the ANC. I met Diana again less than two weeks later. Diana had called me several times to ask me when I would come to see her. When I arrived in her village and called her, she told me she was already waiting for me at the health facility. She invited me to her house and we walked there together in 15 minutes.

Both Diana and her husband come from different regions of Tanzania. They met in this village town in 2007 where they both had moved for work and had family relations. Diana had finished primary school before moving and started to work in a shop with basic groceries. Now she is running her own business of selling second hand clothing. Her husband Alex works as an electrician.

During all her previous pregnancies, Diana went to the same health facility, both for ANC and birth. In 2008, Diana became pregnant for the first time. After 3 months of this pregnancy, Diana got a stomach ache and thick blood loss and decided to go to the health facility for checkup because she realized that there was something wrong.

It was three months (the pregnancy) […] the stomach was having pain […] I was bleeding so I decided to go to the hospital […]at the hospital they wrote me medicine to take from the pharmacy […] so they give medicine yet the pregnancy was gone […] I don’t know what happened (too cause this) but I did work when the blood was coming out so even in this pregnancy they (the nurses) told me not to work.

Three years later, Diana became pregnant for the second time. She was not using any family planning methods and when I asked her if she felt she had to wait a long time before she got pregnant, she said it was just a normal time. During this pregnancy she got malaria, which was diagnosed at a private laboratory. Even though she had already started ANC, when she felt sick she went to the laboratory for a check-up. She remembers that she rested a lot during this pregnancy because of her previous experience. At home people had prepared her for the birth and told her what would happen.

I went with my sister in law […] because I was starting to feel the contractions. Then they took me to the hospital at night. I stayed there, when it was morning, at 9, I started to deliver. I have (a lot of pain), I deal with it anyhow, because it is normal […] you don’t have an option […] I knew what was going to happen, people used to tell me […] they told me when the day of delivery, will happen this and this and then you will go to the hospital […] yaah even the pain they told me […] things to go with (materials you need to bring with you) they had already bought for me.

After birth she started using family planning. She went to a private facility close by her house and chose the intra uterine device. After some time, she was experiencing a foul smell and thick vaginal discharge and decided to go back to the private facility. At the private facility they didn’t have an explanation for the side effects and advised her to take it out. In 2012, quickly after she stopped using the family planning, she became pregnant.

Yes. I went and took it out. It really bothered me, up till now it brings me problems […] They told me; ‘just go to check for these diseases’, I checked for STD’s, I checked for pregnancy, I wasn’t pregnant, but that problem kept on coming back. […] It was November and I took it out, then I got pregnant of her.

Diana still has the ANC card of her previous pregnancy and I saw that she started ANC when she was 5–6 months (22 weeks) pregnant. She visited ANC 2 times during this pregnancy and she delivered in the same facility. It was all a good experience for her without any complications. She had not been using family planning methods since her birth, so when she missed her period for one month she went to the health facility where a urine test confirmed her pregnancy. Her first ANC visit followed soon after.

You have to go (to ANC) to know each and everything what is going on, because when you are not going there you can’t understand what is going on and how the baby is laying in the belly. If you don’t go to the clinic then you don’t know the health of your child.

Like many others she too was confronted with inconveniences and lack of available services at the health facility.

When I am there, I have to wait for long and (when I arrive) the things (services) are not started, so I have to wait long because they did not already start with the work. Sometimes there is one nurse and the women are with many, so we have to wait for a long time. Then you can start to get problems, like stomach, because you didn’t eat since the morning. Because: I wake up in the morning, early in the morning. I left the kids, I went there to the line (waiting area) and I want to be on time. When I was there I was asking myself “I woke up in the morning, I didn’t even prepare for them at home”. So, I am asking myself, “why is it like this?” I am waiting too long.

But, she expressed sympathy for the working conditions of the nurse/midwives, placing responsibility for their insufficient education and lack of resources with the government.

The government should be responsible because they provide medicines or treatment, but they (the nurse/midwives) don’t have it there. When they (the nurse/midwives) don’t have the instruments, you have to go somewhere else […] she (the nurse/midwife) is not responsible for these things.

She knows the limits of the system and if needed seeks care in private laboratories or health centres. During this pregnancy she attended 5 ANC visits. When she was feeling sick, she thought she had malaria and decided to go to a small private laboratory close by her house. There they tested her positive for amoebiasis and a urinary tract infection and wrote her a prescription for medication which she gave to her husband so he could buy it for her.

Sometimes you have to go to the laboratory (private) because if you go the clinic you don’t get service early or sometimes they don’t have instrument … You go to test to know your condition about health condition and health condition of your baby …. When you go to normal one (government facility), some of the tests are not there, or there are a lot of people, you can be there with your pregnancy and stay until two or three so I think it’s better to go even if you pay money but you get the service.

Her strong social network in support of a facility birth made it very likely Diana would give birth again in the health facility also for this pregnancy. She gave birth approximately one month before her due date.

Since Saturday I felt my head was hurting and even the stomach. That night I felt sick, like having a contraction, when I woke up at 6am (on Sunday) I went to the hospital […] I went with my husband […]. When I arrived there (reception of the hospital) I took the card and wanted to get the form (treatment form for sick patients). […] I didn’t expect to deliver that day; I expect to deliver in April. They (at reception) told me to go direct to labour to get measured. […] I went with him (husband) inside there (at the maternity ward), but labour you cannot enter with someone. […]. When they (nurse/midwives) measured me, they told me it was contraction. After telling me that they told me I have to stay there at the hospital, they give the bed to lie there (in the maternity ward). They told me that the way (cervical dilatation) is not yet ready, so they told me to do exercise. So, when I was there I started doing exercise of walking since morning up to night. […] All that time I was alone, the nurse/midwives were doing their things, you just keep walking and when you are tired you come to sleep. […]. So when it was 2 am the contraction was ready, then I told them that I want to go to labour, because the contraction was ready. When I went to the labour at 3am they measured me again and they said “not yet”. Then they told me to stay there [at the labour bed] because it (cervical dilatation) was a little bit ready. So, I stayed there till I delivered at 7am. I was lying, just turning side to the other side. […] I called them when I felt that I have to push. She came and said “not yet, just keep waiting”. That time when the ‘bottle broke’ (water broke) then I started to deliver. When I called her the contraction was read then she started giving me service. (The nurse/midwife told me) the way to lie down, the way to push the baby. After (the baby was born) then she was cleaning the baby then she cut the cord. There is a place they put the baby there. Then they come for me, to clean me. After they finish cleaning me then she gave me the injection.

## Flora (Unexpected facility birth)

I (SP) met Flora for the first time when she entered the ANC room in the health center. Both Flora and Nurse Happy laughed some times while she asked the questions of the ANC card about demographic information and risk factors. Flora answered with a confident and loud voice, made eye contact and looked around to see what was going on. Nurse Jane continued and asked Flora about her previous pregnancies.

They asked me, this is your first pregnancy, I say no, the first pregnancy was twins, then the first child died and the second one died on the next week, so they say ‘pole’ (sorry)

Nurse Jane performed an HIV test and weight measurement. After this, Flora was asked by Nurse Happy to climb on the bed for further examination. Then Flora was invited to sit on the bench. Nurse Happy explained to Flora that she is HIV positive. She spoke with a soft tone of voice, and looked at Flora, explaining about the medication she needed to take, how often and why. She received her medication: a plastic box with HIV medication, the red pills to increase the blood and medication for malaria. Then Flora was told to go the other side of the building to open a file and get registered at the HIV clinic. This took approximately 10 minutes. I asked Flora if I could come with her to her house so I would be able to reach her for an interview since she does not have a phone. She agreed and welcomed me at her house. This time we went with a piki piki (motor cycle taxi) but normally Flora comes by foot to the health facility which takes her up to 1.5 hours.

On foot! […] Because up till the centre in (village name) is 1 hour yes, or maybe and a half […] On foot it’s far, true […] When you don’t have money you walk

After one week I came back to her house for the first interview. Flora is 21 years old. She was born in another district, where she finished standard seven primary education. However, she is unable to read or write. In 2014, when she was 19 years old she became pregnant for the first time. She was still living at her parental home. She was happy with the pregnancy but her parents didn’t like it because she was not married. The boy didn’t want to marry her because he was already married with to another woman.

The first born were twins […] I delivered at home; I was with my mom […] yeah, she told me to push, she collects the thing we cut on the placenta […]one of them cried and the other one opened the mouth and died […]one was still alive for one week, after one week he died.

After one year, in 2015, she met her current husband who came to introduce himself at her parents’ house and they agreed on the marriage. Until now he hasn’t paid the dowry because he is not in a good financial situation and her parents have accepted that. He is originally from this village where they are living now. She moved in with him, together with her younger brother Isaack, who is 5 years as she takes care of him. Her husband works as a fisherman in a town which is 5 hours away by bus. Flora started to work on the land her husband owns and they grow rice and groundnuts. Soon after she moved here she got pregnant with the current pregnancy. Her husband is often away from home but sends money to her

He is a fisherman […] He comes home on Sunday […] I cannot know what he is doing […] I can’t trust him because am not staying with him long time.

During this pregnancy she planned to start ANC at the end of December, when she was around 6 months pregnant. But, the first time she went to the heath facility she was refused and told to come back with her husband. Flora decided to try again one month later in January which is when I met her.

Yes, you are troubled going to the clinic every month, better when you start when there are just a few months left […] It’s better when you go to the clinic for 2 months and you give birth in the month that follows […] When you skip a month they will start insulting you. They’ll refuse to give you a check-up.

Despite repeated information provided to her by the nurse/midwife about her HIV status, Flora refused to accept the result and did not take the recommended treatment

I don’t believe if it is true I have to go back and take the test again. […] yeah, they checked me then they didn’t say anything, they kept quiet […] maybe the test is not ok, the equipment to test […] I and my husband should know how to solve it, but the nurses they should stop (talking about HIV).

Her refusal to accept her HIV status appeared to come from her general distrust of the health services, partly because of inconveniences caused by the long distance to reach the facility, long waiting hours, denial of services and lack of caring behaviour of providers.

If you listen to nurses’ rules, you’ll die with your baby […] She was just angry […] I just experienced their behaviour, the nurses, I don’t want it to happen again.

Flora initially also refused the ‘blue bag’ when this was offered to her. The blue bag was part of a government incentive program to motivate women to give birth at the health facility. Because Flora planned a home birth she did not want to accept the bag.

It (the bag) has rules. You pay a fine even of 10.000 TZS (5 USD) if you use one thing (at home).

Despite Flora’s poor adherence to facility ‘rules’, including refusal to visit the HIV clinic, she faithfully went to her scheduled ANC visits. She said this is expected of every pregnant woman and that ‘only the nurse/midwives know what the condition is of the pregnancy’. Flora explained her only option was to give birth at home.

I can’t get people who can help me there (at the hospital) […] because when you are there you cannot get anybody to do my work here (home). When you find the person to go with you at the hospital, you also need to find someone who can cook for you at home then bring food to the hospital. That’s why I need someone to help with that […] she can help to catch the baby and to remove the sheets […]. I will look for someone maybe I can order someone from my home place. […].

I visited Flora on both the Saturday and Sunday after I had seen her for her last visit in the facility. Her due date had passed so I went to her house to see how she was doing. She told me that now, already for three days, the pain started. ‘The pain comes and goes’, she said. I asked her if she already knew who will help her during the birth and she told me that a neighbour is coming to her house, pointing to the other side of the road.

The following Monday I was surprised to meet Flora at the post-natal ward at the facility, after she had given birth in the hospital.

That day at midnight I woke up and realized that it was labour pains. […] I walked myself to the neighbours to inform them […] I didn’t have anybody to send there. The neighbour is just close by, he has a pikipiki (motorcycle). We (Flora and the neighbour’s daughter Meki, 16y) were dropped there [at the health facility] […]. I asked her (Meki) to see where they (the nurse/midwife) usually sleep […] The man who was driving the pikipiki he knocked (on the door) and then she (nurse/midwife) woke up […]. We went in the room to examine and then she said “the way (cervical dilatation) is too little”. She said “why you tell this person who take you here to go away because you need to go to (another hospital)”. Then she just went and woke up the other doctor. She said “come and examine her”. The doctor said that there is enough way, just to go and sleep (lie down) and just wait for the delivery. Now I went to go and sleep and the pain was continuing so I was sometimes sitting, sleeping like this. And then (at 5am) I sent her (Meki) to go and call the nurse/midwife and then she came and she said’ “it’s still not yet”. When she (the nurse/midwife) finished examining, she said to Meki to sit on the chair and to look at me and then to make sure I am just sleeping on the side and then not to push. And then when the nurse/midwife had left then the water broke. […] At 6am. […] I was just going to continue to pushing now. […] She (Meki) called the nurse/midwife. […] She came and said “start pushing now the head is out”. Then when she was cutting the cord, she gave me the baby […] and then she wrapped the khanga (Tanzanian fabric) around him.

## Maria (Unexpected home birth)

I (SP) met Maria for the first time at the end of April during her first visit. Even though she had welcomed me at her home and explained the directions, I was unable to locate her. When I met her at the health facility during her second visit, I sat down next to her and greeted her. She told me that she was surprised that I had not visited her yet. I told her that I searched for her but could not find her. She laughed and could not believe that is was hard for me to find her house since it is very close to the facility. Around mid-day we walked to her house and it took ten minutes. Maria rents a room on a small compound and lives there by herself.

Maria is originally from another district. In 2008, when she was 14 years old, she became pregnant. By that time, she had just finalized primary school. She married the father of her child and he paid her parents 200,000 Tsh ($100 USD) as a dowry. She delivered her daughter at home without complications. It was her mother giving her support and after asking why she stayed home, Maria said:

Maybe because mothers were delivering at home and we just copy that […] when you don’t have a problem you just give birth […] I feel it is difficult to go to the hospital […] to be ruled by the nurse.

Maria told me about her getting pregnant so young and she expressed to me that she made a mistake, that she just wanted to ‘try one time’ and did not expect to get pregnant. After giving birth, Maria stayed home to look after the house and her child. After some years her husband started to drink and mistreat her.

Ya, I was very young […] We we’re married, then we divorced […] Hate, he was accusing me […] he bothered me.

Maria decided to leave him in 2014 and brought her daughter to her parents’ home. Maria came to Mwanza ‘to look for life’ and stayed with a lady friend of hers. She followed her friend to the current town where she found work as a cleaning lady.

In 2016, Maria became pregnant again with her boyfriend, Charles, who has a small shop for daily groceries. They are not married but see each other regularly. Charles helps her with small things during the pregnancy and visits her once in a while.

We’re just lovers […] He has a shop […] No, I can’t (marry him) because I was already married so I don’t see it has benefit.

Maria was surprised when she found out that she was pregnant. She was using injections as a family planning method, she explained, it might be that she became pregnant because it had been a long time since her last injection.

I didn’t plan to get pregnant […] Ya, the nurse told me not to use amoxicillin, because amoxicillin, it reduces the power of the injection for family planning […] Ya, I feel ok (about this pregnancy), because it already happened so I have to accept it.

Due to the unexpected pregnancy, Mariam was not sure how many weeks pregnant she was and when she could expect to give birth. She told me that she expected her due date to be in July but because the nurses had told her August, so she was not sure.

During this pregnancy Maria had gone to 2 ANC visits. The third one was scheduled but it was on a day she had to work. Maria asked me if I could change the date (for her third visit) on the ANC card to the next month because she expected the nurses to shout at her when they will see that she did not show up on the scheduled date. We agreed to go together on the end of this month and see what will happen.

There is a job which I am doing, it was interrupting in the same date so I didn’t go […] I am not afraid but they will scream at me. Because I am the one, it is my fault, my laziness.

When discussing her plans for birth Maria explained to me that it is important to give birth at the health facility, despite possible difficulties she might face in the hospital.

Ya, it’s my task to go there, I have to […] It’s important to go there, because otherwise what do you do when a problem comes up and you don’t attend any clinic; what do you do. […] It depends on the nurses, when you are lucky you have a gentle nurse. There are others you find them they are just rude, that means she is rude and that’s just how she is.

When I came back to the health facility at the agreed date, two weeks later, I did not see her. When I visited her at home instead, she laughed out loud and showed me her baby. She said the birth had come unexpectedly, and that she did not manage to go to the hospital.

It was Monday, I went to (village name) to do cleaning (her work), after cleaning I went back home here and then because the water was running (from the outside tap) again I was fetching water. Then when it was night I slept. I didn’t know it was contractions because I was just used to the … I get used to this condition of pain in the waist. When the condition was changing I didn’t know it was contractions, I thought it was the same problem of waist. That’s why I didn’t want to call anyone, I thought it would be disturbance because I get used to it. I didn’t know if it was contractions because I didn’t, it was not water or blood that came out, so I didn’t know. When it was 1am I was alone in the room, my boyfriend was not here, he went, he had travelled. When the condition was changing then I didn’t call anyone. Then I took the fabric down (on the floor). Then I sit down (squatting) and then I start to push myself. After (the baby was born) I called the neighbours. […] I called that mama and that lady (pointing at the neighbouring houses). Then they came and called the nurse/midwife (a nurse/midwife who lives nearby). She started to give me service. She cut the cord, she closed the belly (tied a piece of fabric around Maria’s stomach), then she took the baby up on the bed. […] She left immediately. I gave her 5.000 TSH ($2,5) because it’s not good when someone is giving you service and then you don’t give anything. […] She didn’t ask me, I decided myself.

## Discussion

Through the collection of narratives of Tanzanian women’s reproductive pathways, this study sought to increase understanding of how women’s ultimate birth location can be understood by their responses to significant life events including previous pregnancy and birth experiences. The four narratives presented illustrate how women’s decision-making for care seeking and the weight which is given to the range of different factors (e.g previous experiences, distance to facility, availability of resources, social networks) depends on women’s agency, including their perception of self, the self in relation to its social environment and reflection on risks associated with the decision to seek care or not. Women’s life stories and how these were shared revealed women’s personal values, social networks, motivation and adaptive style, surfacing an implicit theory of action, which helps us understand their different birth locations [11]. Women in poor countries are often portrayed as passive and submissive to their surroundings, lacking capacity to act or decide for themselves [^37–39^]. However, within the constraints of their lives, women plan and make choices based on the options available to them [23,37], as all women in this study show. Over the past few decades, and across the continuum of their reproductive lives, women have not been standing still as objects of their pregnancies and as passive recipients of care. Women have learned from their previous experiences, increased their awareness of the range of options they have and through this, embraced the possible challenges they can face in the event of a next pregnancy.

All women in this study were born between 1980 and 2000 and had their first pregnancies between 1995 and 2015. The women in this study, therefore, had different exposure to what they could expect during pregnancy and birth, either through their own -, their mothers’-, or other family members’ experiences. For example, home birth seemed to be the default choice for Maria and Flora’s first births, likely because this is how their mothers gave birth, during a time where home birth was accepted practice. Even though facility birth has been actively promoted, in particular since the year 2000 still today many women view facility birth as necessary primarily for when complications occur [7]. Maria’s life experiences give insight into of a resilient self, dealing with the situations as they appear. Accepting the reality of becoming a mother at the age of 14, but not accepting to remain in an abusive relationship. For Maria pregnancy and birth seemed to ‘just happen’. Even though she planned for care seeking during ANC and birth out of a belief that this was needed ‘in case a problem happens’, when birth was imminent, she dealt with it, there and then, at home.

Timing of women’s pregnancies and the ‘birth order’ is dependent on whether the pregnancy was intended, wanted or just happened [40]. The current pregnancies were very welcome for most women. For Maria and Paulina, however the pregnancy was unintended. Paulina already had 7 children and had not been pregnant for the past 6 years. Maria had no intention of becoming pregnant either, but she was slightly careless with her contraceptive use. Some studies describe that unintended pregnancy can result in poor healthcare seeking for ANC and birth [40,41]. ‘The birth order’, the number of prior births and where they took place provide women with important experiences that influences their choices [6,7]. Women with previous facility birth experiences might fall back on facility births because they had positive previous experiences, such as for Diana. In particular, after experiencing a miscarriage and waiting several years after marriage before getting her first child. With support from her social network, husband and family, she appeared confident in the health system which ensured her she would have a positive outcome. Experiences with care received in facilities however, throughout all of the women’s pregnancies and births, are usually a mixture of both positive and negative experiences [27] at the same time. For Diana her self-efficacy likely made her resilient and capable of dealing with health system constrains. If the health system could not deliver what she needed, she knew she could seek help in private facilities, giving her a range of options.

If women have their own access to resources, their decision-making power increases, which could give potential for facility births [6]. However, Kabakyenka et al. [42] found that if women were the sole decision makers, facility birth was less likely. Maria and Diana both had their own source of income, in addition to their partners’ support, but this did not necessarily lead to facility birth. Women in active labour rely on a social network for support and assistance to effectuate their decisions. Women who moved away from their family homes and did not live close to the families of their husband increasingly relied on support from neighbours. Flora, Maria and Paulina all contacted their neighbours for help when labour started or directly after birth. Women’s narratives show how they also depend on the nurse/midwives in the nearby health facilities for their support when the time comes for birth. Non-supportive behaviour and even instances of disrespect and abuse are increasingly documented and form important disincentives for facility birth [7,27]. Women find ways to cope with sub-standard care and take measures to ensure good rapport with nurse/midwives [43,44]. For example, women sometimes ‘dress up’ to appear more well-of, or give nurse/midwives small gifts, such as Paulina did, as an extra insurance. Maria received help from a health provider after giving birth at home, and ensured payment for services provided. Another way of dealing with poor quality of care is to avoid this care altogether and stay at home. Flora was unable to exercise control over many aspects of her life. Despite Flora expressing that she believed it was best for her to give birth at home, she was unable to do so as her neighbours sent her to the health facility. It is possible that her perceived risk of staying at home without support, including her history of losing her twins, may have far outweighed the risks associated with a facility birth, even if this would mean she had to submit to a system she did not believe in.

The stronger a woman’s self-perception of coping skills, the more willing she is to take risks or embark on unusual courses of action [24]. However, the perception of what constitutes a risk is greatly influenced by how informed women are, past experiences, social positioning including power relations and sense of self-worth [37,45]. From a medical perspective, the risk Paulina took with staying at home, was likely far greater than she herself could have understood. The chances of her getting post-partum haemorrhage were quite high, considering her being a multi-para and having experienced heavy bleeding before. However, from Paulina’s perspective the risk of using her limited funds and the likelihood of not receiving any services at the health facility far outweighed the potential benefits. Paulina also appeared increasingly experienced after several home births, which likely strengthened her confidence to stay at home. Careful consideration of how and when poor women spend their limited resources seems to be very important. Similar findings have been reported elsewhere [37].

If the global community truly strives to ensure skilled care for all, ensuring quality of care in health facilities should be the highest priority. This will function as the primary pull factor for all women. It seems likely that if women believe their efforts are worth it, they will go to a health facility and they will plan for it, no matter the resources, distances or efforts required. As long as health systems favour advantaged groups, be it those who can afford them or those who know how to deal with system constrains, SBA strategies will still result in uneven and inequitable outcomes [37]. In the meantime, as long as women have poor experiences in health facilities, women will strengthen their self-efficacy and exercise agency in their decision to give birth at home. This group of women is not easily ‘tricked’ to come to a health facility though interventions intending to motivate women to seek care, as long as the facility conditions remain the same. At the same time, the group of women that faithfully have facility births despite these poor health system conditions, or do not know any better, risk being exposed to a normalization of sub-standard treatment, including lack of respect for their dignity, which might ultimately influence their perceptions of self-worth.

## Conclusion

Women’s narratives illuminated their individual lived experiences throughout their reproductive pathways until their recent birth. Rich narratives combined with description of women’s previous pregnancy experiences and life events provides increased understanding of the complex individual decision-making processes, within the context of the northern part of rural Tanzania. The role of isolated factors, the weight these are given, and the, often tacit, meanings of perceived risks and opportunities helps us to understand why some women in this study have a facility birth and some do not. Women’s agency and their perceived self-efficacy determine if women feel the power to influence their situation or if they choose to rely on external factors to lead the way. Women in this study appeared resilient and made choices based on their perceived realistic capabilities to seek care. Recognizing the legitimacy of such choices can be a starting point for strengthening individual women’s capacity to influence change. Ultimately demanding health systems capable of delivering high-quality care, while recognizing women’s values and needs, which is essential for women to have positive pregnancy and birth experiences. While no study can provide findings that are universally transferable [34], this study shows the relevance of the voices and narratives of those whose health concerns we are seeking to understand and to analyze, offering a comparative advantage in applying a similar study to other places and contexts beyond Tanzania.

## Paper context

Despite increased access to facilities, many women in Tanzania give birth at home. Determinants for women’s care seeking remain poorly understood. Few studies are able to answer why and how women make their decisions for birth. This study provides increased understanding of this complex decision-making processes, the role of isolated factors and meanings of perceived risks and opportunities for facility or home birth.
